# Insights from the cold transcriptome of *Physcomitrella patens*: global specialization pattern of conserved transcriptional regulators and identification of orphan genes involved in cold acclimation

**DOI:** 10.1111/nph.13004

**Published:** 2014-09-10

**Authors:** Anna K Beike, Daniel Lang, Andreas D Zimmer, Florian Wüst, Danika Trautmann, Gertrud Wiedemann, Peter Beyer, Eva L Decker, Ralf Reski

**Affiliations:** 1 Plant Biotechnology, Faculty of Biology, University of FreiburgSchänzlestraße 1, D-79104, Freiburg, Germany; 2 Institut für Humangenetik, Universitätsklinikum FreiburgBreisacherstr. 33, D-79106, Freiburg, Germany; 3 Cell Biology, Faculty of Biology, University of FreiburgSchänzlestraße 1, D-79104, Freiburg, Germany; 4 Institut National de la Recherche Agronomique28 rue de Herrlisheim, F-68021, Colmar, France; 5 FRISYS - Freiburg Initiative for Systems Biology79104, Freiburg, Germany; 6 BIOSS–Centre for Biological Signaling Studies79104, Freiburg, Germany; 7 FRIAS– Freiburg Institute for Advanced Studies79104, Freiburg, Germany; 8 TIP–Trinational Institute for Plant Research79104, Freiburg, Germany

**Keywords:** abiotic stress, abscisic acid (ABA), cold acclimation, microarray, moss, orphan genes, *Physcomitrella patens*, transcriptome

## Abstract

- The whole-genome transcriptomic cold stress response of the moss *Physcomitrella patens* was analyzed and correlated with phenotypic and metabolic changes.
- Based on time-series microarray experiments and quantitative real-time polymerase chain reaction, we characterized the transcriptomic changes related to early stress signaling and the initiation of cold acclimation. Transcription-associated protein (TAP)-encoding genes of *P. patens* and *Arabidopsis thaliana* were classified using generalized linear models. Physiological responses were monitored with pulse-amplitude-modulated fluorometry, high-performance liquid chromatography and targeted high-performance mass spectrometry.
- The transcript levels of 3220 genes were significantly affected by cold. Comparative classification revealed a global specialization of TAP families, a transcript accumulation of transcriptional regulators of the stimulus/stress response and a transcript decline of developmental regulators.
- Although transcripts of the intermediate to later response are from evolutionarily conserved genes, the early response is dominated by species-specific genes. These orphan genes may encode as yet unknown acclimation processes.

## Introduction

Mosses are representatives of an early diverging lineage of land plants with a haploid-dominant life cycle. Molecular data suggest that the split between mosses and vascular plants occurred at least 470 million yr ago (Mya) during the late Ordovician (Zimmer *et al*., 2007; Lang *et al*., 2010). During this period (488–444 Mya), global climate change included gradual cooling and glaciations, probably caused by the expansion of land plants which reduced the atmospheric CO_2_ content (Lenton *et al*., 2012). Thus, the molecular mechanisms enabling plants to cope with changing climatic conditions must have evolved early in evolution. The potential of mosses to endure such dramatic periods was shown recently by the revival of different species after 400 yr of ice entombment (La Farge *et al*., 2013).

Even today, about two-thirds of the world's landmass is exposed to temperatures below zero (Larcher, 2001; Beck *et al*., 2004). Especially in the temperate zone, plants may acquire freezing tolerance by cold acclimation, a process based on the exposure to low, non-freezing temperatures (Thomashow, 1999; Winfield *et al*., 2010). This process is accompanied by alterations in lipid composition and an accumulation of cryoprotectants and antioxidants to increase the reactive oxygen species (ROS)-scavenging potential of the cells (Thomashow, 1999; Beck *et al*., 2004). A reduction in photo-oxidative stress is achieved, amongst other pathways, by the synthesis and conversion of photoprotective pigments, such as carotenoids (Demmig-Adams *et al*., 1996), together with non-photochemical quenching (NPQ), including photoinhibition (Müller *et al*., 2001).

The gene regulatory networks that contribute to cold acclimation are well described for flowering plants (Chinnusamy *et al*., 2007; Winfield *et al*., 2010), but so far only few large-scale (Sun *et al*., 2007) and no genome-wide data are available for mosses. By contrast with vascular plants, mosses are poikilohydric and rely directly on their environment for water balance. This necessitates a considerable tolerance against dehydration, ranging from the toleration of moderate, short-term dehydration to complete desiccation for longer periods (Oliver *et al*., 2005). As a consequence, mosses must have evolved molecular mechanisms that allow for fast and controlled cessation of metabolic activity as a function of their environment.

Over recent decades, the moss *Physcomitrella patens* has been developed as a plant flagship model organism as it combines numerous advantages for evolutionary and molecular studies (Cove *et al*., 2006; Strotbek *et al*., 2013), and can be cultivated under standardized *in vitro* conditions (Hohe *et al*., 2002). Although *P. patens* does not hibernate in the leafy state, it acquires an increased freezing tolerance either by previous cold acclimation or by abscisic acid (ABA) treatment (Minami *et al*., 2003, 2005; Nagao *et al*., 2005; Bhyan *et al*., 2012). An increased freezing tolerance of gametophores was shown after 48 h of cold (Wang *et al*., 2012), whereas transcriptional studies described transcript accumulation of selected genes during the first hours of cold. Some of these genes also respond to ABA, whereas others do not (Minami *et al*., 2003, 2005; Frank *et al*., 2005; Cuming *et al*., 2007; Sun *et al*., 2007; Bhyan *et al*., 2012). Genome-wide analysis of the cold transcriptome from early signaling events to the initiation of cold acclimation may help to discover as yet uncharacterized genes that contribute to the cold and dehydration tolerance of mosses.

Employing the established experimental setup of the above-mentioned small-scale studies, we present the cold stress response of *P. patens* on the basis of whole-genome microarray experiments. We identified the genes involved in abiotic stress signaling, as well as the initiation of cold acclimation, and resolved temporal relationships covering selected time points between 1 and 24 h of cold. Our data are discussed in an evolutionary context in order to describe principles of cold acclimation shared among land plants, but also to reveal moss-specific gene expression patterns which may have contributed to the evolutionary success of these poikilohydric plants.

## Materials and Methods

### Plant material and cold stress treatments

Gametophores of *Physcomitrella patens* (Hedw.) Bruch & Schimp. were grown on solid medium (250 mg l^−1^ KH_2_PO_4_, 250 mg l^−1^ KCl, 250 mg l^−1^ MgSO_4_·7H_2_O, 1000 mg l^−1^ Ca(NO_3_)_2_·4H_2_O, 12.5 mg l^−1^ FeSO_4_·7H_2_O) (Reski & Abel, 1985) under standardized growth conditions in a climate chamber (16 h : 8 h light : dark period, 70 μmol m^−2^ s^−1^ light intensity, 23°C). The strain used in this study is a subcultivar of the sequenced Gransden 2004 strain stored in the International Moss Stock Centre (No 40001). After 4 wk of growth, cold treatments were performed by transferring the plates to ice in polystyrene boxes, following the procedure described by Minami *et al*. (2005). During treatment, the temperature of the medium was reduced to 3.5°C (± 1°C) within the first hour (Supporting Information Fig. S1a). The treatment was carried out under the same light conditions in the climate chamber. The time points of cold exposure were 1, 3, 8 and 24 h. All gametophores were harvested at the same time of day at 16:00 h, 10 h after the beginning of the light period. The treatment was started at the appropriate time before (Fig. S1b). The control plants were grown in parallel and harvested at the same time as the cold-treated gametophores, and frozen directly in 2-ml Eppendorf tubes in liquid nitrogen. Three replicates from independent plates, cultivated in parallel, were harvested per time point.

### RNA extraction and purification

Total RNA was extracted from frozen gametophores with cetyltrimethylammonium bromide (CTAB) buffer (Chang *et al*., 1993). Before RNA extraction, autoclaved CTAB buffer was mixed with 2% 2-mercaptoethanol and pre-warmed to 65°C in a water bath. One volume of CTAB buffer (3 ml per 500 mg of moss tissue) was added to the plant material, incubated for 10 min at 65°C and mixed in between to lyse the cells completely. After centrifugation (10 min, 2300 ***g***), the supernatant was transferred to a fresh reaction tube and a CHISAM (chloroform : isoamylalcohol, 24 : 1) extraction was performed twice, each followed by a centrifugation (20 min, 2300 ***g***). RNA was precipitated over night with 5 M LiCl, washed with 70% ethanol and subsequently diluted in nuclease-free water. For the microarray experiments, the RNA was purified with an RNeasy Kit (Qiagen, Hilden, Germany) with on-column DNA digestion using DNAse I according to the manufacturer's protocol.

### Quantitative real-time polymerase chain reaction (qPCR)

For qPCR, cDNA was synthesized using the TaqMan® Reverse Transcription Reagents Kit (Applied Biosystems, Darmstadt, Germany) according to the manufacturer's protocol with random hexamer primers. For each of the three technical replicates, cDNA corresponding to 50 ng of total RNA was used per transcript to be quantified. A non-transcribed (-RT) control was included to confirm correct DNase digestion. Primers were designed using the Roche Universal Probe Library Assay Design Center (https://www.roche-applied-science.com). The primers for qPCR are listed in Table S1. Melting curve analysis was performed for each primer pair before further analyses. The real-time PCR was performed with the SensiMix™ Kit and SYBRGreen (Bioline, Luckenwalde, Germany) in a LightCycler® 480 (Roche, Mannheim, Germany). Normalization of variations in cDNA content was performed with the genes EF1α (Pp1s84_186V6.1) and C45 (Pp1s107_181V6.1). Both reference genes are not cold responsive. The relative abundance of transcript levels was calculated relative to the reference genes according to Livak & Schmittgen (2001). Each qPCR experiment was performed in at least three biological replicates.

### Microarray hybridization

The microarray experiments were performed with a 90 K whole genome microarray (Combimatrix Corp., Mukilteo, WA, USA) and a probe design as described previously (Wolf *et al*., 2010). In total, 1.5 μg of RNA were applied for each time point per biological replicate, reverse transcribed into cDNA and amplified into amplified RNA (aRNA). Subsequently, 5 μg of aRNA were labeled with Cyanin-5 (RNA ampULSe: amplification and labeling kit; Kreatech, Amsterdam, the Netherlands). The resulting labeled aRNA was fragmented (Fragmentation Reagents; Ambion, Austin, TX, USA) and hybridized overnight onto the microarray following the manufacturer's instructions. Visualization was performed with a laser scanner (Genepix 4200A; Molecular Devices, Ismaning, Germany) and images were analyzed with the Microarray Imager 5.9.3 Software (Combimatrix Corp.). Each time point was repeated in three biological replicates. The microarray slides were stripped with a stripping kit (Combimatrix Corp.) and reused up to four times. The experimental procedure was the same as that described previously (Richardt *et al*., 2010; Wolf *et al*., 2010).

### Large-scale gene expression profiling

Transcriptomic data were statistically analyzed with Genedata Analyst® Version 7.0 (www.genedata.com). The loaded raw data were median normalized. For statistical analysis of the gene expression levels, a K groups analysis with implemented ANOVA (Clarke & Cooke, 1998) was performed to identify differentially expressed genes (DEGs). The false discovery rate (FDR) was determined with a Benjamini–Hochberg (BH) correction (Benjamini & Hochberg, 1995). The genes for further analysis were selected according to a BH *P* value of < 0.05. For comparison in pairs of the chosen time points, a Tukey's HSD (honestly significant difference) *post hoc* test for one-way ANOVA with Bonferroni multiple testing correction was applied. Genes with a Bonferroni-corrected *P* value of < 0.05 were selected for each comparison in pairs. An effect size analysis was performed to determine the fold change means for each comparison in pairs. Microarray data are available in the ArrayExpress database (www.ebi.ac.uk/arrayexpress) under accession number E-MTAB-2165.

### Annotation and phylogenomics analysis of DEGs

For the DEGs, the gene ontology (GO) (Ashburner *et al*., 2000) annotation of the recent genome annotation (Zimmer *et al*., 2013) was analyzed with BLAST2GO. A Fisher's exact test was performed to provide an overview on over- and under-represented GO terms, divided into the categories ‘biological processes’, ‘cellular components’ and ‘molecular function’. The phylogenomics analysis was published by Zimmer *et al*. (2013), comprising all-vs-all protein homology clustering of 27 sequenced Archaeplastida species. These gene family definitions were used to assess the phylogenetic context and to assign gene family annotations of the DEGs, especially focusing but not limiting to the (co-)orthologs in *Arabidopsis thaliana*. In order to infer detailed orthology relationships, selected gene families were further characterized by reconciled phylogenetic inference based on mapped cDNA-alignments (MAFFT L-INSI of protein sequences) using PHYML/treebest and a dated species tree (Lang *et al*., 2010). Table S2 provides the list of (co-)orthologs for each of the DEGs according to Zimmer *et al*. (2013).

### Annotation, classification and analysis of transcription-associated proteins (TAPs)

Previously classified TAP families from *A. thaliana* and *P. patens* (Lang *et al*., 2010) were combined and categorized into GO biological process classes ‘developmental process’ (GO:0032502), ‘response to stress’ (GO:0006950) and ‘response to stimulus’ (GO:0050896) based on the TAIR (http://www.geneontology.org/GO.downloads.annotations.shtml) and cosmoss.org (https://www.cosmoss.org/physcome_project/wiki/Downloads) GO annotation releases. The resulting sets of TAP-coding genes were used to tabulate the family members into the classes ‘developmental process’, ‘response to stimulus or stress’ and ‘developmental process and response to stimulus or stress’. The resulting tables were used to determine significant association of TAP families with each of the classes using Fisher's exact test with correction for multiple testing using the BH method at 95% confidence. Not all TAP families have sufficient experimental evidence to allow robust statistical classification. However, all families were classified into the three categories, resulting in three different types of annotations: quality class 1 supported at 95% using FDR-corrected *P* values, class 2 at 95% based on non-adjusted *P* values, and class 3 which are solemnly based on the selection of the process class with the maximal number of annotated family members. The resulting classifications were used to analyze the fold changes of the TAP-coding transcripts along the time course using generalized linear models (GLMs). All steps were performed using a combination of custom bash, Perl and R scripts.

### Extraction and analysis of carotenoids

Tissues were lyophylized and ground (Dismembrator MM301, Retsch, Haan, Germany). Around 20 mg of lyophilized material was taken up in 10 ml acetone : 100 mM Tris (8 : 2, v/v); 100 μg *of* α-tocopherol acetate (Sigma-Aldrich, Deisenhofen, Germany) was added as internal standard (Schaub *et al*., 2005), mixed by vortexing, sonicated (Sonifier 250; Branson, Danbury, CT, USA) and centrifuged to separate insoluble material. The supernatant was transferred to a fresh Falcon™ tube. The supernatants of two additional acetone extractions were combined with the first. A phase separation was achieved with light petroleum : diethylether (2 : 1, v/v) and H_2_O. The organic phase was collected, dried and dissolved in 1 ml of chloroform. For high-performance liquid chromatography (HPLC), a Waters 2695 Separations Module (Eschborn, Germany) equipped with a Waters 2996 photodiode array detector was used. The separation was performed using a YMC-Pack C_30_-reversed phase column (250 × 4.6 mm i.d., 5 μm; YMC Europe, Schermbeck, Germany) with the solvent systems B (methanol : *tert*-butylmethyl ether : water (60 : 12 : 12, v/v/v)) and A (methanol : *tert*-butylmethyl ether (500 : 500, v/v)). The column was developed at a flow rate of 1 ml min^−1^ with a gradient from 100% B to 43% B within 45 min, then to 18% B within 20 min, followed by a ramp to 0% B and 100% A within 2 min. At this point, the flow rate was increased to 2 ml min^−1^ maintaining 100% A for 9 min. Finally, the column was re-equilibrated with 100% B and a flow rate of 2 ml min^−1^ for 10 min. Carotenoids were identified by their absorption spectra, monitored by the photodiode array detector and applying the max plot option from 300 to 550 nm. All peaks were normalized relative to the internal α-tocopherol acetate standard. An unpaired *t*-test was performed with the QuickCalcs online *t*-test calculator (http://www.graphpad.com/).

### Quantitative analysis of ABA

Gametophores were ground in liquid nitrogen and lyophilized; 30 mg were extracted for 1 h using an overhead shaker at room temperature with 1 ml of 80% acetone + 1% acetic acid. Samples were spiked with 50 ng of (±)-2-*cis*,4-*trans*-ABA-d6 (D6-ABA; Icon Isotopes, Summit, NJ, USA). After centrifugation (20 000 ***g***, 5 min), the supernatant was collected, the extraction was repeated for 15 min and the supernatants were combined. The samples were evaporated for removal of the organic solvent (Concentrator-plus; Eppendorf, Hamburg, Germany). The aqueous phase was acidified with HCl and partitioned three times against ethyl acetate. The combined epiphases were dried and re-dissolved in 40 μl of methanol, 2 μl of which was injected. Separation was performed using a Dionex Ultimate HPLC system (Thermo Fisher Scientific, Dreieich, Germany) equipped with a 1.9-μm Hypersil Gold C18 reversed-phase column (150 × 2.1 mm; Thermo Fisher Scientific). A gradient system was used, consisting of solvent A (0.05% (v/v) aqueous acetic acid) and solvent B (0.05% (v/v) acetic acid) in acetonitrile. The gradient consisted of a linear segment from 100% A to 100% B in 8 min. The final condition was maintained for 4 min, all at a flow rate of 0.4 ml min^−1^. Targeted high-resolution mass spectrometry was carried out using a qExactive spectrometer (Thermo Fisher Scientific) equipped with a heated electron spray ionization (HESI) source operated in negative ion mode. Spectra were recorded using a spray voltage of 3.5 kV and a capillary temperature of 350°C. Nitrogen sheath gas flow, auxiliary gas flow and sweep gas flow were set to 40, 10 and 2 arbitrary units, respectively. Full MS1 spectra and targeted MS2 spectra were recorded using a normalized collision energy of 20%. Internal standard-based quantification was performed using the MS data and Tracefinder 3.1 software (Thermo Fisher Scientific). The exact mass of ABA (*m/z *− 1 = 263.12888) and the MS2 daughter ions (*m/z* = 219.13866 and 204.11307) were used for identification. The unlabeled authentic reference (±)-ABA was from Sigma-Aldrich.

### Pulse-amplitude-modulated fluorometry

*In vivo* chlorophyll fluorescence of gametophores that were exposed to cold for 1, 3, 8 and 24 h at 70 μmol m^−2^ s^−1^ light intensity was measured with a FluorCam 800 MF (Photon Systems Instruments, Drasov, Czech Republic). Control plants were grown at 23°C. The measurements were performed after 40 min of dark adaptation at room temperature. Saturating light was set to 30%; actinic light was set to 70%. Data analysis was performed with the software PSI FluorCam, Version 0.5.2.18 (Photon Systems Instruments). The compared values were the maximum photosystem II (PSII) quantum yield in the dark-adapted state calculated from the variable fluorescence *F*_v_ and the maximal fluorescence *F*_m_ of the dark-adapted state (*F*_V_/*F*_m_), and non-photochemical quenching in light (NPQ_Lss).

### Microscopy

Stereomicroscopy was performed with the Stemi 2000C (Zeiss, Jena, Germany). Images were taken with a charge-coupled device camera (AxioCam MRc5 and ICc1; Zeiss) and scaled with AxioVision software 4.8 (Zeiss). For scanning electron microscopy (SEM), gametophores were fixed on a holder and frozen in slush nitrogen. In the prechamber of the scanning electron microscope (Philips XL30 ESEM), the samples were warmed to −80°C and sputtered with 20 nm gold. The SEM images were taken at −150°C.

## Results

### Temperature reduction results in dehydration, bleaching and inhibition of growth

**Fig 1:**
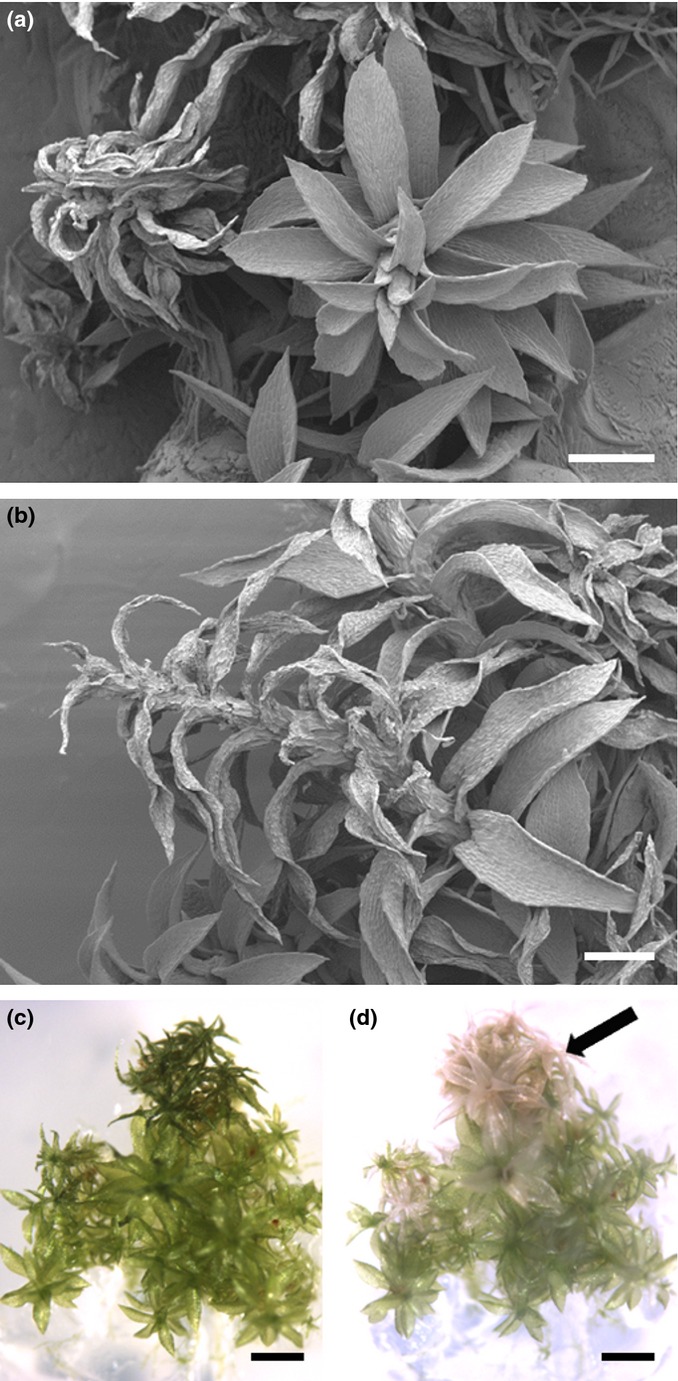
Temperature reduction causes dehydration of *Physcomitrella patens* gametophores. (a) *Physcomitrella patens* gametophores exposed to cold dehydrate when grown on solid medium. (b) Dehydration starts from the tip of the gametophore. (c) Desiccation during cold exposure caused bleaching of the dehydrated tissue after a few days (d). (a, b) Scanning electron microscopy of gametophores exposed to low temperature for 24 h; images were taken at the Microscopy Center of the University of Basel (ZMB); (c) 4 d cold-exposed colony with dehydrated gametophores; (d) chlorotic gametophores 1 wk after the cold treatment (right, black arrow). Bars: (a, b) 300 μm; (c, d) 1 mm.

Gametophores grown on solid medium were exposed to cold by transferring the cultivation vessels to ice. Subsequently, gametophores dehydrated, starting with very few plants after 8 h and affecting approximately one-quarter of the gametophores after 24 h. Dehydration was only visible in some gametophores within a colony (Fig.1a) and started from the tip (Fig.1b). Desiccation (Fig.1c) was lethal, as evidenced by bleached gametophores after 1 wk (Fig.1d).

The filamentous protonema was strongly affected by temperature reduction, resulting in an inhibition of growth (Fig. S2a) and development (Fig. S2b). Although control cultures developed caulonema, buds and gametophores, the cold-exposed protonema was completely inhibited in development, showing chlorotic and roundish cells (Fig. S2b), resembling brachycytes, which are a typical cellular response of mosses to ABA-mediated stress responses (Decker *et al*., 2006). Although growth inhibition was not quantified in gametophores, the transcriptomic data provide evidence for an inhibition of growth and for an involvement of ABA during the cold response.

### The cold transcriptome of *P. patens*

**Fig 2:**
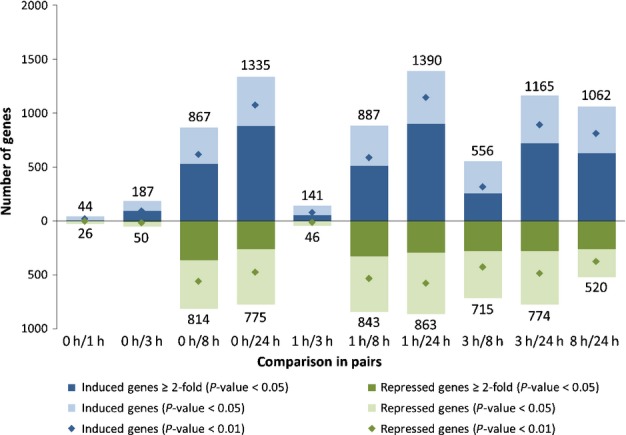
Differential gene expression in response to cold stress. Significantly differentially expressed genes of *Physcomitrella patens* in response to cold were determined with an ANOVA, followed by a *post hoc* test for comparison in pairs. Statistical analyses were performed with Analyst 7.0 software (www.genedata.com). Significantly induced genes for the comparisons in pairs (blue); significantly repressed genes (green). Differentially expressed genes with a Bonferroni-corrected *P* value of < 0.05 (3220 genes, light blue and light green). All genes with at least two-fold induction (1987 genes, dark green and dark blue). Genes with a more stringent *P* value cut-off of 0.01 (3004 genes, diamonds).

The cold transcriptome was characterized via whole-genome microarray analyses. The selected time points for cold exposure were 1, 3, 8 and 24 h. Differential gene expression was analyzed relative to a control (0 h) grown under standard temperature (23°C). During the entire time course, 3220 genes were significantly differentially expressed (BH-corrected *P* value of < 0.05). Hence, at 95% confidence, the expression of 11.6% of the genes represented on the microarray was affected by cold (Table S3). Of the 3220 DEGs, transcripts of 1987 accumulated or declined at least two-fold (Table1). When comparing the number of DEGs between the control and the four time points, respectively, an increase in DEGs over time was evident, starting from 70 DEGs after 1 h to 2110 DEGs after 24 h of cold stress (Fig.2). The DEGs for each time point in comparison with the control are listed in Tables S4–S7. Furthermore, the mean values of the transcript abundance fold change increased over time (Table1).

**Table 1:** Number of differentially expressed genes and gene expression fold changes during the time-series of cold stress of *Physcomitrella patens*

Comparison in pairs	Number of differentially expressed genes (induced/repressed)	Number of differentially expressed genes, ≥ 2-fold change (induced/repressed)	Range of gene expression fold change (mean)
0 h/1 h	70 (44/26)	6 (6/0)	0.35–7.92
0 h/3 h	237 (187/59)	101 (93/8)	0.24–11.44
0 h/8 h	1681 (867/814)	839 (529/364)	0.07–31.95
0 h/24 h	2110 (1335/775)	1142 (883/259)	0.05–127.79
1 h/3 h	187 (141/46)	55 (53/2)	0.34–10.68
1 h/8 h	1730 (887/843)	842 (514/328)	0.76–39.51
1 h/24 h	2253 (1390/863)	1195 (902/293)	0.61–190.66
3 h/8 h	1271 (556/715)	532 (254/278)	0.09–16.69
3 h/24 h	1939 (1165/774)	1001 (721/280)	0.04–38.44
8 h/24 h	1582 (1062/520)	891 (629/262)	0.07–21.76

**Fig 3:**
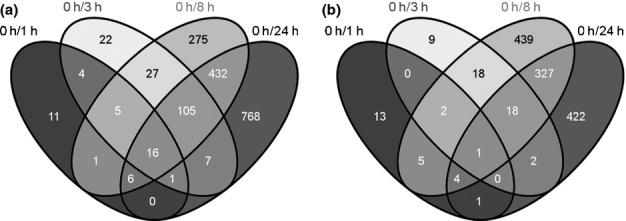
Comparison in pairs of differentially expressed genes in *Physcomitrella patens*. All significantly induced (a) and repressed (b) genes derived from pairwise comparisons were compared with each other. The Venn diagrams depict the overlaps between each pairwise comparison. Venn diagrams were constructed with Venny (Oliveros, 2007).

For all comparisons in pairs, a large number of genes were specific for a certain time point (Fig.3). Among all DEGs, transcripts of 16 genes over-accumulated persistently (Fig.3a) and one declined persistently (Fig.3b). Two of the persistently over-accumulating transcripts encode AP2 (APETALA2)/EREBP (ethylene-responsive element binding protein) transcription factors (Pp1s373_18V6.1, Pp1s38_257V6.1), whereas the persistently declining transcript encodes a MIKC*-type MADS box transcription factor (Pp1s163_70V6.1/Pp1s163_69V6.1). Furthermore, two transcripts potentially encoding calcium ion binding proteins (Pp1s168_83V6.1) and a heat shock protein (HSP) (Pp1s174_35V6.1) over-accumulated persistently (Table S8).

### Validation of gene expression profiles

**Fig 4:**
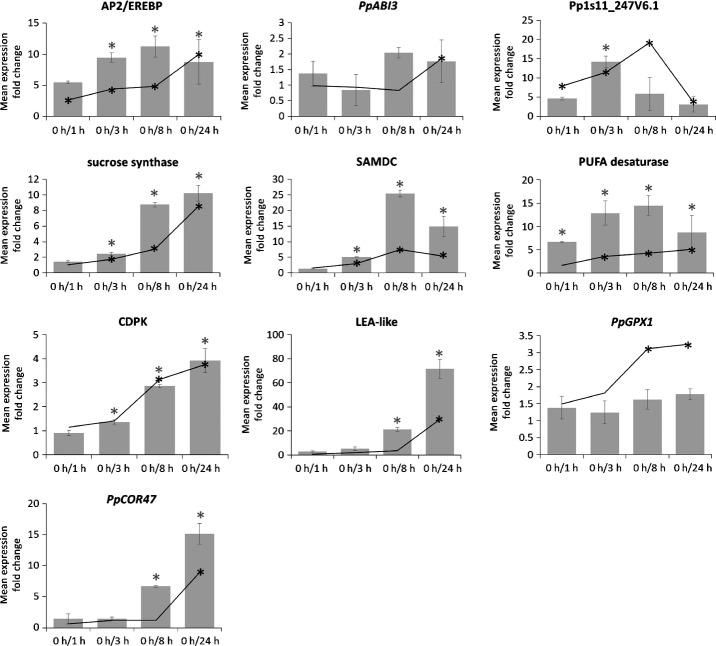
Validation of gene expression patterns of *Physcomitrella patens*. Selected expression profiles were validated with quantitative real-time polymerase chain reaction (qPCR). Two genes encode transcription factors (AP2/EREBP, APETALA2/ethylene-responsive element binding protein: Pp1s373_18V6.1, *PpABI3b*; abscisic acid insensitive 3: Pp1s173_143V6.1). Further genes encode a sucrose synthase (Pp1s93_98V6.1), an *S*-adenosylmethionine decarboxylase (SAMDC, Pp1s335_22V6.1), a putative polyunsaturated fatty acid desaturase (PUFA desaturase, Pp1s286_53V6.1), a glutathione peroxidase (GPX, *PpGPX1*: Pp1s98_2V6.1) and a calcium-dependent protein kinase (CDPK, Pp1s309_91V6.1). A late embryogenesis abundant (LEA)-like protein-coding gene (Pp1s267_21V6.1) and the dehydrin-coding gene *PpCOR47* (cold-responsive, Pp1s442_22V6.2) were late induced. Gray bars, mean fold changes of expression obtained from qPCR for at least three biological replicates. Error bars represent the standard error of the mean. Black lines, means of expression fold changes from microarray analyses. Asterisks show significant induction (black, microarray; gray, qPCR): *, *P* < 0.05.

The expression profiles of 10 DEGs, spanning different aspects of the cold response from signaling to the initiation of cold acclimation, were independently analyzed by qPCR (Fig.4). One of these transcripts encodes an AP2/EREBP transcription factor (Pp1s373_18V6.1), which accumulated early and persistently. The transcription factor-coding gene *PpABI3b* (ABA insensitive 3, Pp1s173_143V6.1, Marella *et al*., 2006) was exclusively late induced after 24 h of cold exposure, but not significantly according to qPCR. One gene without annotation (Pp1s11_247V6.1) and without detectable orthologs in other Archaeplastida was induced early, being the transcript with the highest over-accumulation after 1 and 3 h of cold (Tables S4, S5). Further genes encode a sucrose synthase (Pp1s93_98V6.1), an *S*-adenosylmethionine decarboxylase (SAMDC, Pp1s335_22V6.1), a fatty acid desaturase (Pp1s286_53V6.1) and a calcium-dependent protein kinase (CDPK) (Pp1s309_91V6.1). The induction of the glutathione peroxidase (GPX)-coding gene *PpGPX1* (Pp1s98_2V6.1, Frank *et al*., 2007) was only significant in the microarray. A late embryogenesis abundant (LEA)-like protein-coding gene (Pp1s267_21V6.1) was amongst the highest over-accumulating transcripts after 24 h (Table S7). The expression pattern of the dehydrin-coding transcript *PpCOR47* (Pp1s442_22V6.2, Frank *et al*., 2005) was also confirmed (Fig.4). Taken together, this independent evaluation revealed a high quality and reproducibility of the microarray-derived expression patterns.

### GO annotation and classification

For all DEGs, GO term annotations (Zimmer *et al*., 2013) were analyzed; 2558 of the 3220 DEGs were annotated in at least one of the three GO categories ‘cellular component’, ‘biological process’ and ‘molecular function’, whereas 662 (20.5%) had no available GO annotation (Table S3). Pairwise gene set enrichment analyses using Fisher's exact test was performed to search for over- and under-represented GO terms (0 vs 1 h/3 h/8 h/24 h). The datasets of the early time points in comparison with the control yielded no significantly over- or under-represented GO terms, whereas the datasets of the intermediate and late time points in comparison with the control yielded numerous significantly over- and under-represented GO terms (Table S9). Based on this, the transcriptomic data were divided into three groups, representing early (1, 3 h), intermediate (8 h) and late (24 h) responses.

### Characterization of the gene regulatory response to cold

**Fig 5:**
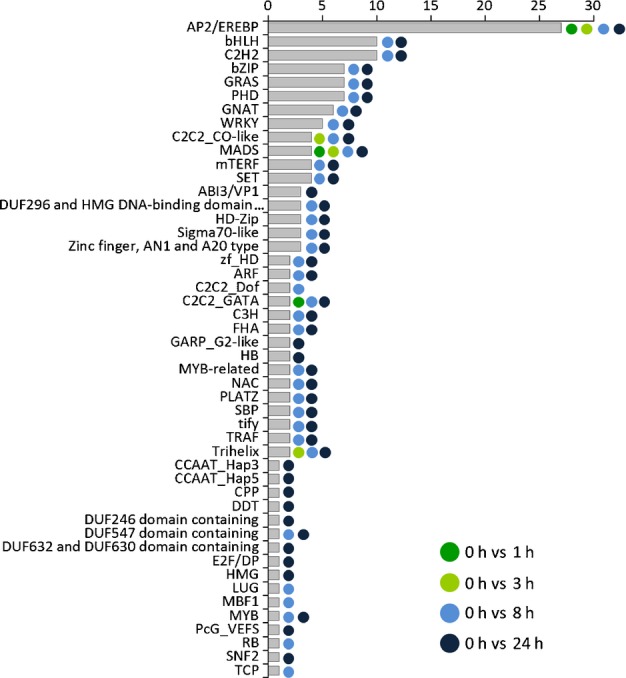
Differentially expressed transcription-associated protein (TAP)-coding genes in *Physcomitrella patens*. During the time course of cold stress, 156 genes belonging to 48 TAP families were differentially expressed when comparing the analyzed time points with the control (0 h). The absolute number of differentially expressed genes (DEGs) belonging to the TAP family is shown on the *x*-axis. The TAP families according to Lang *et al*. (2010) are listed on the *y*-axis. The color code shows the differential expression of genes belonging to a certain TAP family for that time point in comparison with the control.

In total, 156 TAP-coding genes belonging to 48 of the total of 96 TAP families (Lang *et al*., 2010) were differentially expressed during the time course (Fig.5). Consideration of all pairwise comparisons resulted in 172 differentially expressed TAP-coding genes belonging to 48 families (Table S10). Their number increased over time, starting from four DEGs after 1 h to up to 114 DEGs after 24 h (Table S10). Over-represented TAP families include the AP2/EREBP with 27 DEGs, basic helix–loop–helix (bHLH) with 10 DEGs and the zinc finger proteins C2H2 with 10 DEGs, in comparison with the control (0 h) (Fig.5).

Based on GO annotations and TAPScan gene family assignments (Lang *et al*., 2010), all TAP families of *A. thaliana* and *P. patens* were classified according to their responsiveness to external stimuli/stress and their involvement in the regulation of developmental processes. This revealed 34 TAP families as preferentially stress responsive and 20 families as preferentially involved in developmental regulation, whereas two families (AUX/IAA and MIZ-type zinc finger) did not show signs of specialization (Tables S11, S12). The inferred process classification was used to classify and distinguish cold-dependent gene regulatory patterns of stimulus- or stress-responsive transcriptional regulators, as well as those of developmental regulators. The global comparison of the mean fold changes using GLM analysis indicated a significant induction of stimulus/stress-responsive TAPs (*P*-value = 2.11e-05) throughout the time course, whereas developmental regulators were significantly repressed (*P* value = 0.00808). GLM analysis of the individual comparisons between the time points and the control (0 vs 1 h/3 h/8 h/24 h) demonstrated a well-orchestrated regulatory pattern. Stimulus/stress-responsive TAPs were induced throughout the entire time course (*P *= 0.0262/0.000263/3.79e-05/0.00195), whereas the repression of developmental regulators was restricted to the early and intermediate phases (0 vs 3 h, *P* value = 0.040270; 0 vs 8 h, *P* value = 0.00311).

The GLM analysis was also used to identify regulatory trends of individual TAP families. AP2/EREBP (*P* value = 0.0306) and C2C2 Dof (*P* value = 0.0144) families were preferentially induced throughout the time course, whereas HD-Zip, MYB, TIFY, AN1- and A20-type zinc finger proteins were induced specifically after 8 h. We found no evidence of time point-specific or global repression of individual TAP families.

### The early cold response

After 1 h of cold, 70 DEGs were detected (Table S4). Most over-accumulating transcripts encode two putative mitochondrial substrate carrier family proteins (Pp1s232_9V6.1, Pp1s232_12V6.1), a persistently induced AP2/EREBP transcription factor (Pp1s373_18V6.1), a calmodulin (Pp1s168_83V6.1) and two putative proteins without detailed GO annotation (Pp1s11_247V6.1, Pp1s100_144V6.1). After 3 h, the number of DEGs increased to 237 (Table S5). With regard to the biological processes after 3 h, GO annotation provides evidence for the beginning of metabolic changes, indicated by the GO term ‘biosynthetic process’ (GO:0009058). For instance, carbohydrate metabolism, especially sucrose and starch metabolism, was affected. Early changes measurable at transcriptomic level encompass the induction of α- and β-amylase-coding genes (Pp1s223_59V6.1, Pp1s121_168V6.1), as well as a sucrose synthase-coding gene (Pp1s93_98V6.1). Furthermore, the metabolism of amino acids changed during cold, indicating increased polyamine (spermine and spermidine) synthesis, as transcripts coding for an arginine decarboxylase (Pp1s41_319V6.1), SAMDC (Pp1s335_22V6.1) and a spermine/spermidine synthase (Pp1s279_58V6.1) accumulated after 3 h (Table S5). DEGs coding for polyunsaturated fatty acid (PUFA) desaturases (Pp1s98_209V6.1, Pp1s286_53V6.1) and chalcone synthases (Pp1s22_4V6.1, Pp1s426_47V6.1) indicate the need for the regulation of the membrane's physicochemistry and of flavonoid biosynthesis. Moreover, the early response encompassed the accumulation of HSP- (Pp1s91_206V6.1, Pp1s297_5V6.1, Pp1s204_80V6.1) and LEA-like protein-coding transcripts. LEA-like protein-coding transcripts (Pp1s370_29V6.1, Pp1s51_40V6.1) were amongst the most over-accumulating after 3 h of cold.

### Photoprotection during cold

The intermediate and late phases of the cold response were characterized by increasing oxidative stress, photoinhibition and photoprotection. Numerous transcripts coding for components of photosystem I (PSI) and PSII declined significantly (Tables S6, S7). Correspondingly, over-represented GO terms of the sets of declining transcripts after 8 and 24 h encompassed, among others, ‘plastid’ (GO:0009536), ‘photosynthesis’ (GO:0015979) and ‘photosystem’ (GO:0009521) (Table S9). In addition, various light-harvesting complex (LHC) protein-coding genes were differentially expressed. Although chlorophyll *a*–*b* binding (CAB) protein-coding transcripts declined, RNAs encoding another class of LHC proteins, namely early light-induced proteins (ELIPs), accumulated (Tables S6, S7).

**Fig 6:**
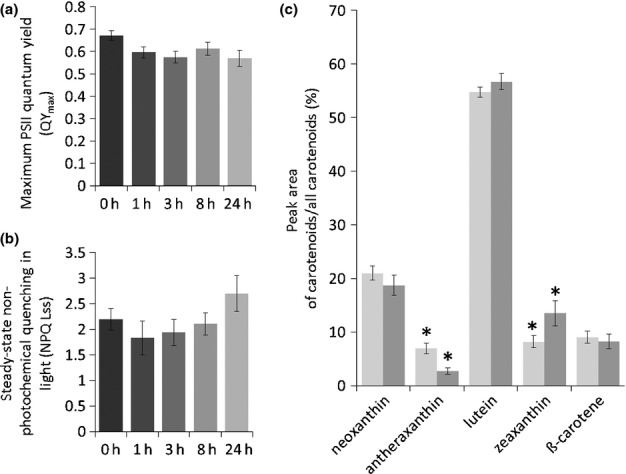
Changes in the maximum photosystem II (PSII) quantum yield, non-photochemical quenching in light and the carotenoid profile in response to low temperature in *Physcomitrella patens*. (a, b) Pulse-amplitude-modulated chlorophyll fluorescence measurement (PAM) of cold-treated gametophores. (a) The maximum PSII quantum yield (QY_max_) is slightly decreased in response to cold stress for 1, 3, 8 and 24 h. (b) Non-photochemical quenching in light (NPQ_Lss) is slightly increased after 24 h at low temperature. PAM was performed in biological replicates (*n* ≥ 3); the error bars show the standard error of the means. (c) After 24 h of cold stress, the carotenoid profile of gametophores changed significantly in comparison with the control. The carotenoids neoxanthin, antheraxanthin, lutein, zeaxanthin and β-carotene are shown in order of their retention times. The peak area of each carotenoid relative to the peak area of all carotenoids is shown: light gray bars, results from gametophores grown under standard conditions; dark gray bars, results from 24-h cold-treated gametophores (*n* = 3, error bars show the standard deviation). Significant changes are marked by asterisks: the amount of the xanthophyll antheraxanthin is significantly decreased (*, *P* = 0.0036), whereas the xanthophyll zeaxanthin is significantly enriched (*, *P* = 0.0256).

In order to monitor the photosynthetic activity of *P. patens* during cold stress, the maximum PSII quantum yield (QY_max_) and non-photochemical quenching in light (NPQ_Lss) were determined with pulse-amplitude-modulated chlorophyll fluorescence measurement (PAM). During cold stress, QY_max_ was slightly decreased (Fig.6a), whereas NPQ_Lss was slightly increased (Fig.6b). As NPQ depends on accessory pigments, such as carotenoids (Demmig-Adams *et al*., 1996), carotenoid profiles from untreated and 24 h cold-exposed gametophores were established by HPLC (Fig.6c). According to this, the level of zeaxanthin was increased significantly after cold exposure (*P* value = 0.0256). Correspondingly, two putative β-carotene hydroxylase-coding transcripts (Pp1s20_24V6.1, Pp1s373_23V6.1) accumulated after 8 h (Table S6), and one additional β-carotene hydroxylase-coding RNA (Pp1s19_96V6.1) after 24 h (Table S7).

### ABA-dependent gene expression and ABA accumulation

An involvement of ABA in cold acclimation was indicated by various DEGs that had previously either been shown or were predicted to be ABA responsive (Richardt *et al*., 2010; Timmerhaus *et al*., 2011). The overlap of DEGs in response to cold and genes predicted to be ABA responsive increased over time from four DEGs (5.8%) after 1 h to 17.5% of all DEGs after 24 h (Table S13). Further evidence was provided by the over-represented GO term ‘response to abscisic acid stimulus’ (GO:0009737) for accumulating transcripts after 24 h (Table S9). Indeed, ABA levels increased in gametophores in response to cold (Fig. S3). Although ABA levels were low in the controls (Fig. S3a), they increased by 2.5-fold after 24 h and 3.9-fold after 48 h of cold treatment (Fig. S3b). The decreased amount of antheraxanthin after 24 h of cold (*P* value = 0.0036) (Fig.6c), together with the accumulation of a 9-*cis*-epoxycarotenoid dioxygenase (NCED)-coding transcript (Pp1s412_7V6.1) after 8 and 24 h, provides evidence for a *de novo* ABA synthesis upon cold treatment.

### Differential expression of orphan genes

Among all DEGs, a total of 390 (12%) did not cluster with any other Viridiplantae gene according to a previous phylogenomics analysis (Zimmer *et al*., 2013) and were considered to be orphan (Tautz & Domazet-Lošo, 2011), that is species specific (Table S14).

During the first hour of cold, the expression of 14 orphan genes altered (nine induced, five repressed), accounting for 20% of all induced and 19% of all repressed genes for the first time point. After 3 h, 40 orphan genes (34 induced, six repressed) were differentially expressed, accounting for 18% of all induced and 12% of all repressed genes. During these early time points, the relative amount of orphan DEGs is significantly higher (*P* value = 1.05E-02) than during the intermediate and late responses (Table S15). Furthermore, the relative amount of orphan genes is significantly higher (*P* value = 0.006) in the early response in comparison with the intermediate and late DEGs (Table S15). The proportion of orphan genes in the set of induced genes did not differ from the proportion in the set of repressed genes (*P* value = 0.514).

Among the 16 persistently accumulating transcripts, the relative amount of species-specific genes was high, with five (31.25%) of the 16 genes (Pp1s174_35V6.1, Pp1s456_30V6.1, Pp1s11_247V6.1, Pp1s373_18V6.1, Pp1s204_54V6.1). Although two of these genes are annotated as HSP (Pp1s174_35V6.1) and AP2/EREBP transcription factor (Pp1s373_18V6.1) coding, the remaining three have no available annotation (Table S8). In total, 157 of the differentially expressed orphan genes have no annotation (40.2%).

## Discussion

### Molecular mechanisms of cold acclimation

In this work, *P. patens* was used as a model to study the early responses to cold on the transcriptomic level in comparison with phenotypic and metabolic changes. By analyzing a time series of microarray experiments between 1 and 24 h of cold, we found three waves of gene activity, an early, intermediate and a late response, that had profoundly different characteristics. In total, 11.6% of the genes represented on the microarray were differentially expressed during the time course. This number is in the range of numbers reported from *A. thaliana*, which vary between 4% and 20% DEGs depending on the duration of cold and the statistical analysis (Hannah *et al*., 2005; Lee *et al*., 2005; Winfield *et al*., 2010).

For *P. patens*, an increased freezing tolerance on previous cold exposure, that is cold acclimation, has been reported (Minami *et al*., 2005). In gametophores, an increased freezing tolerance was measured after 48 h of cold (Wang *et al*., 2012). Cold acclimation requires the perception of temperature reduction, followed by signal transduction via second messengers, such as calcium, leading to the induction of cold-responsive genes (Beck *et al*., 2007; Winfield *et al*., 2010). According to our analyses, signal transduction was represented, amongst others, by the differential expression of 172 TAP-coding genes from 48 TAP families, as well as genes encoding calmodulins and CDPKs. Furthermore, the cold transcriptome indicates changes in metabolism, including the accumulation of cryoprotectants such as sucrose, antioxidants such as carotenoids and flavonoids, and an increased synthesis of PUFAs and cell-protective proteins. These osmoprotectants and PUFAs have previously been identified biochemically in *P. patens* (Erxleben *et al*., 2012; Beike *et al*., 2014). The accumulation of sucrose synthase-coding transcripts indicates the important role of sucrose during the cold acclimation of mosses (Rütten & Santarius, 1992; Nagao *et al*., 2005). Likewise, polyamines may contribute, as indicated by the accumulation of transcripts encoding an SAMDC and a spermidine-synthase. Both enzymes are involved in polyamine biosynthesis (Alcázar *et al*., 2011).

A major source of ROS formation is the photosynthetic apparatus (Triantaphylidès & Havaux, 2009). In order to reduce photo-oxidative stress, plants possess a variety of mechanisms, including photoinhibition and NPQ. The decline in transcripts encoding core components of PSI and PSII, as well as CAB proteins, together with a decreased PSII quantum yield (QY_max_), indicate cold-induced photoinhibition, whereas evidence for increased NPQ is provided by the increased amount of zeaxanthin and the accumulation of transcripts coding for β-carotene hydroxylases, which are key enzymes of zeaxanthin biosynthesis (Walter & Strack, 2011). Supported by increasing NPQ, we conclude that zeaxanthin-dependent photoprotection occurs during the cold acclimation of *P. patens*. Furthermore, the accumulation of ELIP-coding transcripts reveals their photoprotective role in mosses, as shown previously for *A. thaliana* (Hutin *et al*., 2003).

In this work, we showed that ABA levels increase during cold exposure in gametophores. In addition to the direct measurements, the reduced amount of antheraxanthin after 24 h of cold stress, together with the accumulation of an NCED-coding transcript, point to *de novo* ABA synthesis. ABA plays an important role in stress tolerance of plants by acting as an endogenous messenger, especially with regard to the cellular water balance (Christmann *et al*., 2006). Considering the observed dehydration of gametophores during cold, ABA synthesis might be related to cellular water loss in combination with reduced temperatures. By contrast, Minami *et al*. (2005) found no evidence for ABA accumulation in cold-treated protonema. This may be a result of the different physiological responses of both developmental stages (Decker *et al*., 2006) and/or the different amounts of protective metabolites in both tissues (Erxleben *et al*., 2012; Beike *et al*., 2014). However, we have described ABA-mediated gene silencing in *P. patens* protonema previously (Khraiwesh *et al*., 2010), which is in accordance with the data provided here.

Because the majority of DEGs representing these processes have orthologs in other Viridiplantae, we conclude that basic molecular mechanisms of cold acclimation, ranging from the accumulation of cryoprotectants, antioxidants and protective proteins to photoprotection and ABA-dependent signaling, may occur in the gametophyte of mosses and the sporophyte of flowering plants.

### Specialization of transcriptional regulators and gene regulatory response

Cold acclimation of *P. patens* relies on the modulation of specific transcriptional regulatory networks, as demonstrated by the analysis of the GO and TAP annotations. The complements of transcriptional regulators have expanded substantially during the course of evolution. These expansions have enabled the specialization and modularization of gene regulatory networks to coordinate the responses to external stimuli, as well as developmental programs (De Smet & Van de Peer, 2012; Yamasaki *et al*., 2013).

The comparative analysis of individual TAP gene families suggests that transcriptional regulators are often specialized in either the response to external stimuli, such as, for example, the AP2/EREBP transcription factors of the c-repeat-binding factor (CBF) and dehydration-responsive element binding (DREB) subfamilies in terms of cold and drought stress (Medina *et al*., 2011), or in the regulation of developmental programs, such as MIKC*-type MADS box transcription factors (Gramzow & Theissen, 2010). To elucidate whether these observations can be generalized, we analyzed the TAP families of *A. thaliana* and *P. patens* and classified them according to their importance for the regulation of either stimulus/stress responses or developmental processes. We found evidence for a global specialization of TAP families, as we identified specialization of 20 TAP families in the regulation of developmental processes and 34 families in stimulus/stress response in the two species. In particular, the AP2/EREBP, bZIP, HSF, MYB, TIFY and WRKY families are specialized in the regulation of the cellular responses to external stimuli. The data for specialization in developmental processes are less clear. This can be explained by considering the experimental approaches used to study the respective regulators. The determination of a regulator's involvement in a certain developmental process, for example, by single gene mutant phenotype analysis, requires greater experimental effort than the physiological assays which can be carried out as large-scale transcriptional profiling experiments. Most of the stimulus/stress GO annotations are based on the latter type of experimental setup. Our comparative TAP classification provides a useful guide for the design of future experiments to study members of these families.

Considering cold-responsive TAPs, GLM analysis revealed an antagonistic global expression pattern, namely the transcript accumulation of transcriptional regulators of the stimulus/stress response and the transcript decline of developmental regulators in response to cold. This pattern is consistent with the phenotypic observations for the protonema stage, where an inhibition of growth and development occurred. The persistently declining MIKC*-type MADS box transcript is a prominent representative for developmental inhibition. In *A. thaliana*, MIKC*-type MADS box proteins are involved in gametophyte development (Gramzow & Theissen, 2010) and a comparable role for mosses was postulated (Zobell *et al*., 2010).

As in flowering plants (Medina *et al*., 2011), the AP2/EREBP TAP-coding genes play a dominant role in the global transcriptional cold response in *P. patens*. In *A. thaliana*, one well-characterized signaling regulon of cold acclimation is CBF/DREB, which mediates the differential expression of *c*. 12% of the cold-responsive genes and contributes to cold acclimation (Fowler & Thomashow, 2002; Medina *et al*., 2011). Considering *P. patens*, AP2/EREBP TAP-coding genes were over-represented among the DEGs and their transcripts accumulated along the entire time course. However, clear orthologs of the CBF/DREB key regulators from *A. thaliana* could not be identified by high-throughput phylogenomics relying on full-length protein sequences. A more specialized, detailed phylogenetic reconstruction with multiple methods, coupled with a phylogenetic comparative analysis of expression data from more species, would be required to address the question of AP2/EREBP diversification and specialization. Therefore, although it is the most parsimonious hypothesis, it remains speculative as to whether or not a common signaling regulon was already present in the last common ancestor of mosses and flowering plants.

### Orphan genes: key regulators of cold acclimation?

In total, 12% of cold-responsive genes in *P. patens* have no orthologs in other Viridiplantae. This is a similar rate as described previously for the entire genome (Zimmer *et al*., 2013). Strikingly, the relative amount of these orphan genes was significantly increased to 20% in the early transcriptomic response, indicating that this early cold response may be species or lineage specific, whereas, as discussed above, genes contributing to later occurring metabolic changes are largely conserved among land plants. Considering also the large amount of orphan genes among the persistent DEGs, we conclude an important role of species- or lineage-specific genes in the acquisition of abiotic stress tolerance in moss. To answer the question of whether these genes are related to poikilohydry and hold the key for stress tolerance requires further investigations, for example via targeted gene replacement (Hohe *et al*., 2004). The analysis of novel (not yet analyzed) genes from mosses may also help to improve the resilience of crop plants in a changing climate.

## Supporting Information

**Fig. S1**Cultivation temperature decrease during cold treatment and harvesting scheme of *Physcomitrella patens*. **Fig. S2** Growth inhibition of *Physcomitrella patens* protonema during cold treatment. **Fig. S3** Quantitative abscisic acid (ABA) measurements of *Physcomitrella patens* gametophores.

**Table S1** Primers used for quantitative real-time polymerase chain reaction (qPCR)

**Table S2**List of (co-)orthologs for the differentially expressed genes **Table S3** Differential gene expression **Table S4** Early differentially expressed genes 0 vs 1 h **Table S5** Early differentially expressed genes 0 vs 3 h **Table S6** Intermediate differentially expressed genes 0 vs 8 h **Table S7** Late differentially expressed genes 0 vs 24 h **Table S8** Persistently induced and repressed genes **Table S13** Abscisic acid (ABA)-responsive differentially expressed genes **Table S14** Differentially expressed orphan genes

**Table S9**Fisher's exact test

**Table S10**Differentially expressed transcription-associated protein-coding genes **Table S11** Transcription-associated protein-coding gene classification **Table S12** Quality class annotation

**Table S15** Orphan gene statistics
