# Supplementary material for: Insights from the cold transcriptome of Physcomitrella patens: global specialization pattern of conserved transcriptional regulators and identification of orphan genes involved in cold acclimation
Source: New Phytol. 2014 Sep 10;205(2):869–81. doi: 10.1111/nph.13004 (PMC4301180; doi:10.1111/nph.13004)
Supplement: Supplementary file 1 — Fig. S1Cultivation temperature decrease during cold treatment and harvesting scheme of Physcomitrella patens. Fig. S2 Growth inhibition of Physcomitrella patens protonema during cold treatment. Fig. S3 Quantitative abscisic acid (ABA) measurements of Physcomitrella patens gametophores. [file nph0205-0869-sd1.docx]

**Supporting Information Figs S1-S3**

**
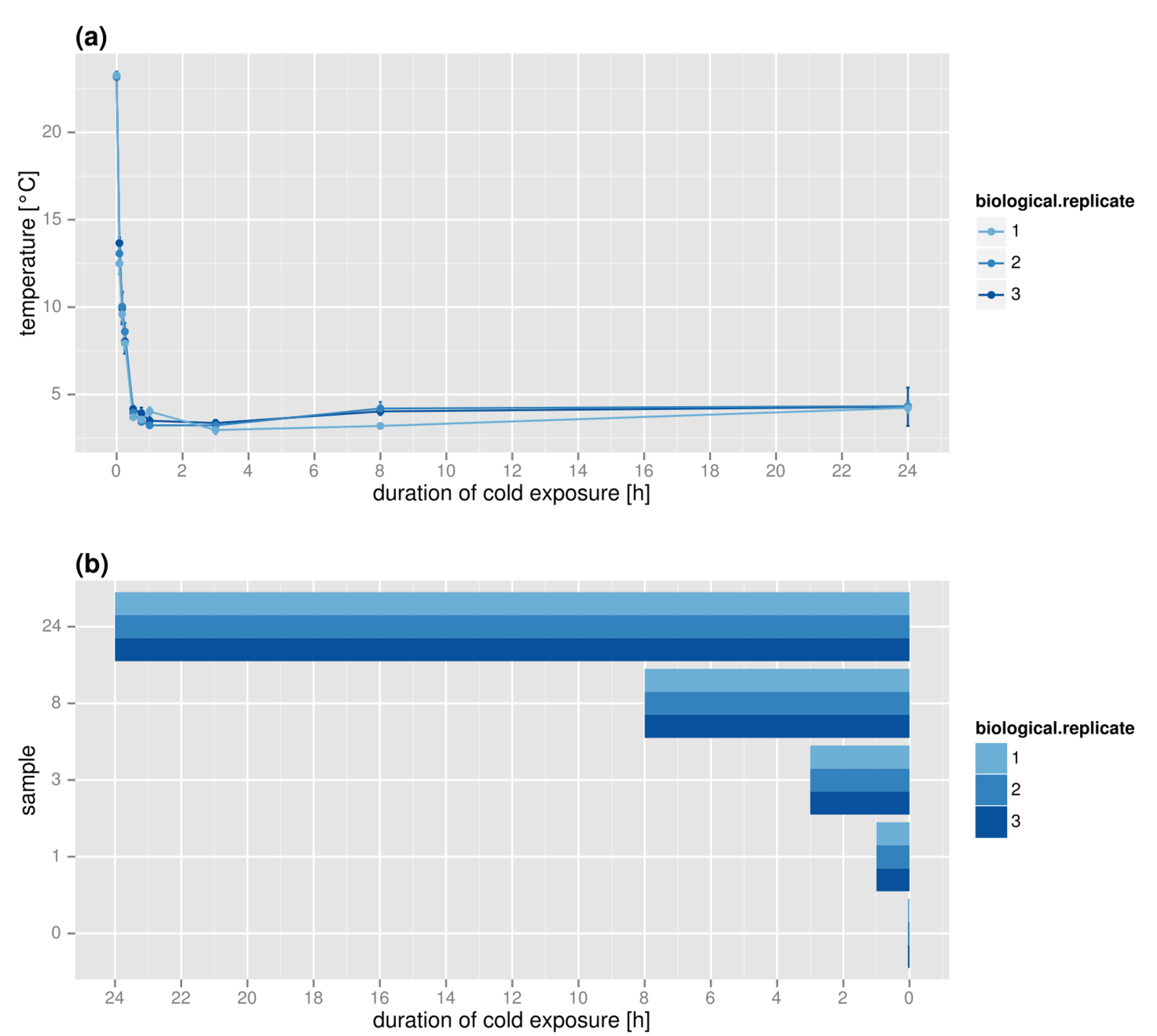
**

**Fig. S1** Cultivation temperature decrease during cold treatment and harvesting scheme of *Physcomitrella patens.* (a) The cultivation temperature decreases during cold treatment from initial 23°C indicated with the blue lines to 3.5°C ± 1 °C. The temperature stays constantly low during cold treatment of *Physcomitrella patens* over three biological replicates. Error bars show the SE. (b) All plants were harvested at the same time and the cold treatment was started the appropriate time before. Blue bars show the three biological replicates per analyzed point in time.

**
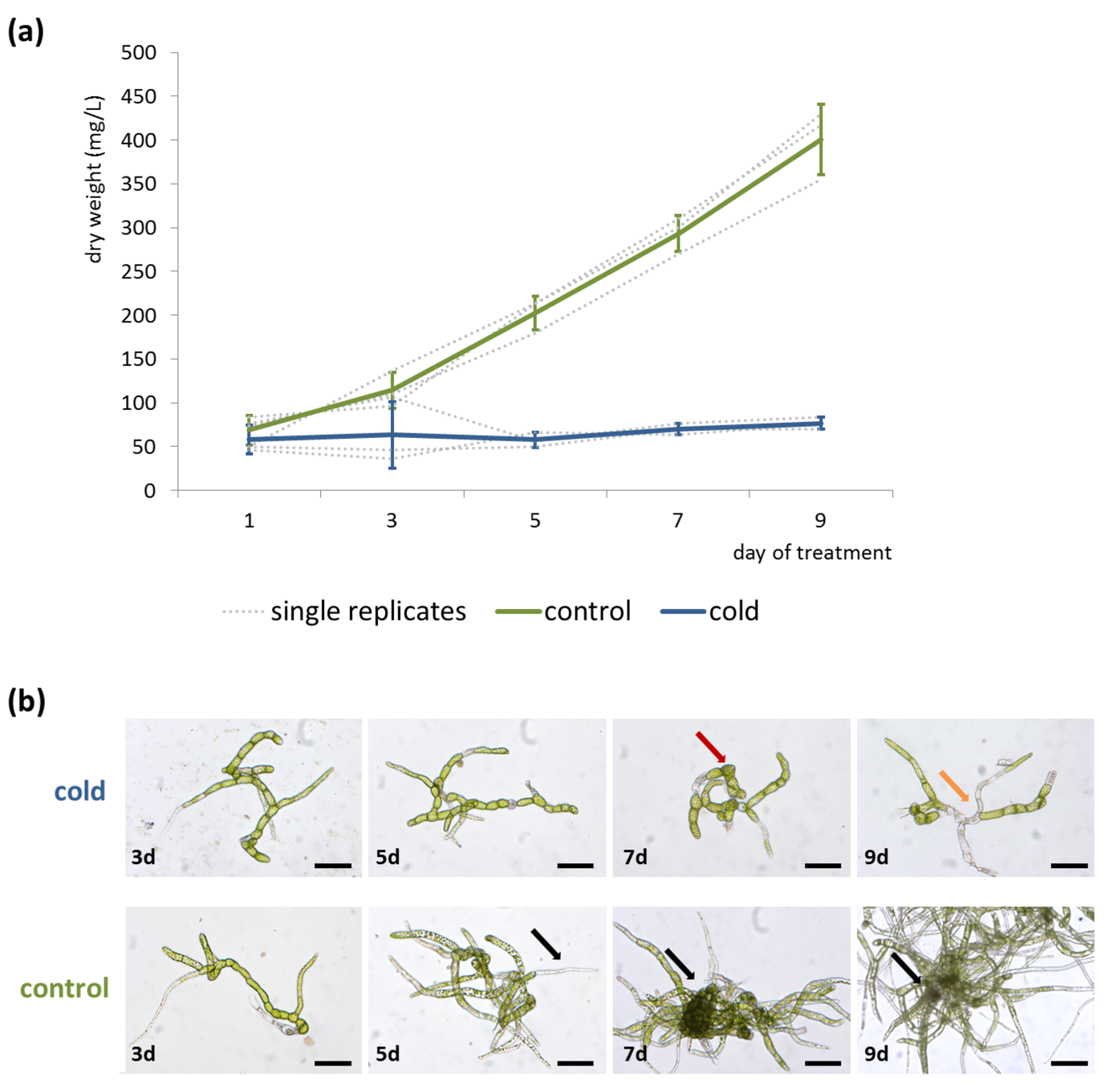
**

**Fig. S2** Growth inhibition of *Physcomitrella patens* protonema during cold treatment. *Physcomitrella patens* protonema was strongly affected by cold stress. (a) The growth of protonema cultures grown under standard conditions of 23°C (control) and under low temperature 3.5 ± 1°C (cold) was determined via dry weight measurement in three replicates (*n*=3, grey dotted lines). While the control shows an increase of biomass over time (green), the cold treated protonema cultures were strongly growth inhibited (blue). (b) Microscopy after 3, 5, 7, and 9 d unravelled also developmental inhibition of the cold exposed cultures. While the control cultures developed caulonema, buds and young gametophores (black arrows), the cold treated cultures arrested in the chloronema stage and developed roundish brachycyte-like structures ( red arrow) and chlorotic cells (orange arrow). Bars, 100 µm.

**
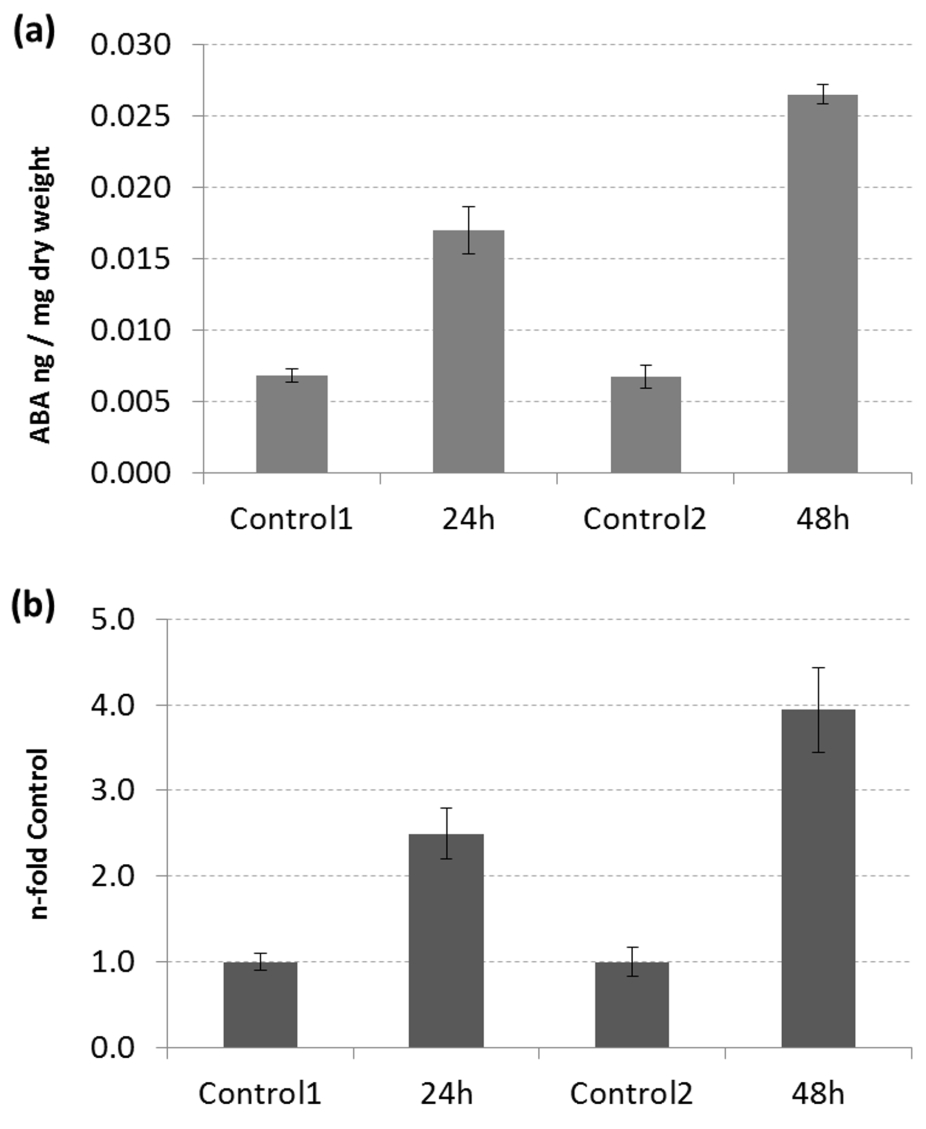
**

**Fig. S3** Quantitative abscisic acid (ABA) measurements of *Physcomitrella patens* gametophores. *Physcomitrella patens* gametophores were exposed to 24 h and 48 h of cold. The controls were grown in parallel under standard conditions at 23°C. (a) This graph shows the absolute content of ABA (ng) per dry weight (mg) of gametophores. At the *x*-axis the sample is shown, at the *y*-axis the absolute content of ABA is shown. The levels of ABA were increased after 24 h and 48 h in comparison to the controls, respectively (*n* = 4, error bars show the SD). (b) This graph depicts the fold change of ABA contents relative to the controls (*n* = 4, error bars show the SD).
